# Comprehensive Multimodal Diagnostics and Rehabilitation in Persistent Post-Concussion Syndrome Following Motor Vehicle Collision

**DOI:** 10.14740/jmc5378

**Published:** 2026-10-02

**Authors:** Kenneth Jay, Mary Shaya, Jonathan Walker

**Affiliations:** a Department of Research, Ethos Health Group, Ocala, FL, USA; b Department of Functional Neurology, Carrick Institute, Cape Canaveral, FL, USA; c Department of Research, Cervello A/S, Roskilde, Denmark; d Department of Medicine, University of Central Florida, Orlando, FL, USA

**Keywords:** Mild traumatic brain injury, Persistent post-concussion syndrome, Videonystagmography, Quantitative electroencephalography, Pupillometry, Vestibular rehabilitation, Multimodal assessment, Motor vehicle collision

## Abstract

Motor vehicle collisions (MVCs) are among the most common mechanisms of mild traumatic brain injury (mTBI), and a meaningful proportion of affected individuals develop persistent post-concussion syndrome (PPCS) characterized by prolonged multidomain dysfunction. Conventional neuroimaging frequently fails to capture the underlying pathophysiology, leaving clinicians without objective diagnostic evidence. We describe a 57-year-old woman who sustained mTBI in a rear-end MVC in March 2023. Head computed tomography (CT) performed at the emergency department and a subsequent brain magnetic resonance imaging (MRI) with susceptibility-weighted imaging (SWI) and diffusion tensor imaging (DTI) were both unremarkable. Despite normal structural neuroimaging, comprehensive multimodal functional assessment identified functional abnormalities across eight domains: vestibular function (videonystagmography (VNG)), cortical electrophysiology (quantitative electroencephalography (qEEG)), pupillary autonomics (automated infrared pupillometry), postural stability (computerized dynamic posturography (CDP)), oculomotor control (saccadometry), cardiac autonomic regulation (24-h heart rate variability (HRV)), olfaction (University of Pennsylvania Smell Identification Test (UPSIT)), and computerized concussion assessment (C3 Logix). Standardized psychological assessments confirmed moderate depression (Patient Health Questionnaire-9 (PHQ-9) = 14), moderate anxiety (Generalized Anxiety Disorder-7 (GAD-7) = 12), clinically significant post-traumatic stress disorder (PTSD) symptoms (PTSD Checklist for Diagnostic and Statistical Manual of Mental Disorders, Fifth Edition (DSM-5) (PCL-5 = 38)), and substantial headache impact (Headache Impact Test-6 (HIT-6) = 58). The patient underwent a 10-modality individualized rehabilitation program. Concussion Symptom Checklist (CSC) scores declined from 92 at initial presentation to 0 by July 2023 (17 weeks post-injury), with near-complete re-resolution to 8 by February 2024 following a transient stress-related exacerbation. When conventional neuroimaging is unremarkable, comprehensive multimodal functional testing may provide evidence consistent with mTBI pathophysiology and help guide targeted rehabilitation. This case illustrates the potential value of a systematic, multimodal approach in PPCS management, while acknowledging that definitive attribution of findings to the index injury is constrained by the single-case design and absence of a pre-injury functional baseline.

## Introduction

Mild traumatic brain injury (mTBI), commonly referred to as concussion, is responsible for an estimated 1.5–3.8 million emergency presentations annually in the United States, with motor vehicle collisions (MVCs) representing one of the leading mechanisms of injury [1]. Although the majority of individuals recover within weeks, a clinically significant subset develops persistent post-concussion syndrome (PPCS), defined as the persistence of cognitive, somatic, and neuropsychiatric symptoms beyond 3 months post-injury [2]. The reported prevalence of PPCS ranges from 15% to 30% of mTBI cases, and the condition carries substantial personal and socio-economic burden [2].

At the cellular level, the pathophysiology of mTBI unfolds as a neurometabolic cascade set in motion by the mechanical forces of impact. Within hours of injury, ionic flux, excitotoxicity, mitochondrial dysfunction, and axonal stretching create a state of neurophysiological vulnerability that may persist for weeks to months [3, 4]. Diffusion tensor imaging (DTI) studies demonstrate white matter microstructural abnormalities in concussed patients that evolve over time and correlate with symptom severity [1, 4, 5], while neurovascular unit dysfunction contributes to prolonged cerebral blood flow dysregulation [6].

The neural networks most reliably implicated in mTBI are the default mode network (DMN), the salience network, the frontoparietal executive control network, and the vestibulo-cerebellar circuits [7–9]. Disruption of these networks produces the characteristic symptom clusters of PPCS: cognitive slowing, attentional deficits, vestibular instability, autonomic dysregulation, and mood disturbance [10, 11]. The neurological examination of reflex dysfunction (NERD) model provides a structured framework for understanding how reflex circuit dysfunction across these distributed networks manifests as the multidomain symptom profile characteristic of PPCS [12].

Standard structural neuroimaging, specifically computed tomography (CT) and conventional magnetic resonance imaging (MRI), regularly fails to capture the microstructural and functional changes that underlie PPCS, a gap that contributes to diagnostic uncertainty and can inadvertently invalidate patient experience [3, 13]. Advanced modalities such as DTI, functional MRI, and quantitative electroencephalography (qEEG) have demonstrated greater sensitivity to the functional disruption characteristic of PPCS [3, 11], but their use in routine clinical practice remains limited.

There is growing consensus that PPCS is best managed through multimodal, interdisciplinary rehabilitation programs that target the specific deficit profile of the individual patient [14–19]. Evidence supports the integration of vestibular rehabilitation, oculomotor therapy, cognitive rehabilitation, autonomic modulation, and nutritional support within a coordinated clinical framework [14, 16, 20].

## Case Report

### Patient demographics and mechanism of injury

A 57-year-old woman was seen at a specialized TBI rehabilitation clinic on March 23, 2023, 2 days after sustaining a rear-end MVC. She was a restrained driver and reported that her vehicle was struck from behind at moderate speed while stopped at a traffic light. She did not lose consciousness. At the emergency department, she was diagnosed with concussion and musculoskeletal strain; head CT was unremarkable, and she was discharged with standard concussion precautions. Her relevant medical history included hypertension managed with medication; she reported no prior concussion, neurological disorder, vestibular condition, or psychiatric diagnosis.

### Initial presentation and symptom profile

At her first clinic visit, the patient described a broad constellation of symptoms. Her Concussion Symptom Checklist (CSC) total score was 92 out of a possible 132, reflecting severe burden across headache, cognitive fogginess, fatigue, dizziness, nausea, photophobia, phonophobia, sleep disturbance, emotional lability, and neck pain domains. She reported that symptoms had been present continuously since the collision and were interfering substantially with activities of daily living and occupational function.

### Diagnostic evaluation

#### Standard neuroimaging

Brain MRI with susceptibility-weighted imaging (SWI) and DTI sequences was performed at an outpatient imaging center. Multisequence T1 and T2-weighted images were obtained. The SWI sequences were negative for microhemorrhage. Whole-brain DTI analysis demonstrated no areas of reduced fractional anisotropy, with the white matter tracts appearing within normal limits throughout. It is important to note that negative DTI sequences do not exclude the clinical diagnosis of mTBI, and a negative brain MRI does not rule out PCS, which remains a clinical diagnosis requiring clinical correlation. Cervical spine MRI demonstrated mild multi-level degenerative disc disease without cord compression. Lumbar spine MRI was unremarkable. Chest and lumbar spine radiographs were negative for acute fracture or dislocation.

#### Computerized Concussion Assessment (C3 Logix)

On March 29, 2023, computerized concussion assessment was performed using the C3 Logix platform (version 3.5.8; Ethos Health St. Pete, device Ocala_Spine_1002), 6 days after the initial clinic visit. The assessment was self-administered and was completed in 27 min and 35 s. This platform integrates a standardized assessment of concussion (SAC), a modified balance error scoring system (mBESS), cognitive processing speed, reaction time metrics, and a graded symptom checklist, and is designed as a concussion screening and monitoring tool rather than a blood or serum assay.

The SAC total score was 22/30 (normative reference: 26/30), with the most pronounced deficit in concentration (1/5), alongside intact orientation (5/5), reduced immediate memory (12/15), and borderline delayed memory (4/5). Simple reaction time was 351 ms (normative reference: 283 ms) and choice reaction time was 590 ms (normative reference: 430 ms), both indicating slowed cognitive processing speed. Processing speed was 50 correct responses (normative reference: 56). The mBESS total error count was 8 (normative reference: 13 on this platform), with single-leg balance on eyes-closed stance generating six errors. Visual acuity line difference was 1.0 (normative reference: 1.8). The graded symptom checklist generated a severity total of 92 (normative reference: 2), consistent with the CSC recorded at initial presentation. The patient’s overall clinical rating on this assessment was 80. Symptoms worsened with both physical and mental activity.

It should be noted that the diagnostic characteristics of this computerized platform, including its sensitivity, specificity, and predictive values for PPCS in adult populations, are not fully established in the peer-reviewed literature, and the normative databases used are proprietary to the C3 Logix system. The findings are therefore interpreted as supportive clinical data within the broader multimodal assessment rather than as independently validated diagnostic benchmarks.

#### Quantitative electroencephalography and brain timing assessment

A 19-channel qEEG recording was performed and compared against a normative database. The normative databases used for this comparison are proprietary to the assessment platform, which should be noted when interpreting the findings. The assessment identified the following abnormalities: a P300 event-related potential voltage of 4.0 µV (normative range: 6.0–12.0 µV), indicating slowed cortical information processing; increased delta power in the frontal and temporal regions; and reduced alpha coherence between frontal and parietal sites. Brain timing assessment (BTA) demonstrated prolonged latency in auditory and visual processing circuits. These electrophysiological findings are consistent with diffuse cortical dysfunction and slowed neural transmission, patterns that have been associated with PPCS in the literature [11]. As with the other modalities in this battery, established sensitivity, specificity, and predictive values for these qEEG parameters in PPCS have not been fully characterized.

#### Videonystagmography

A comprehensive videonystagmography (VNG) battery covering 11 vestibular and oculomotor subtests showed dysfunction in eight of those 11 subtests. Specific findings included reduced gain on smooth pursuit tracking bilaterally; saccadic dysmetria with increased latency; abnormal optokinetic nystagmus; positive Dix-Hallpike test on the right; abnormal cervical vestibular evoked myogenic potential (cVEMP) responses bilaterally; abnormal ocular vestibular evoked myogenic potential (oVEMP) responses bilaterally; and abnormal rotary chair phase and gain metrics. The pattern is consistent with central vestibular dysfunction involving pontine, midbrain, cerebellar, and parietal pathways. Formal sensitivity and specificity data for this VNG protocol in PPCS populations are not available in the published literature for all subtests administered.

#### Pupillometry

Automated infrared pupillometry demonstrated bilateral abnormalities: prolonged constriction latency (right: 248 ms, left: 251 ms; normative range: 180–230 ms); reduced constriction velocity (right: 1.2 mm/s, left: 1.1 mm/s; normative range: 1.5–3.0 mm/s); and reduced maximum constriction amplitude (right: 18%, left: 17%; normative range: 25–40%). These findings indicate autonomic dysregulation affecting the parasympathetic pupillary control pathway. Pupillometric parameters in this range have been associated with brainstem involvement in mTBI [11], although it should be acknowledged that this measure is influenced by age, systemic medication use, and autonomic state at the time of testing.

#### Oculomotor assessment

Computerized oculomotor tracking yielded dysfunctional composite scores in all three domains assessed: pursuits score 60 (normative range: 85–115); saccades score 40 (normative range: 85–115); and fixation stability score 55 (normative range: 85–115). These deficits are consistent with impairment of cerebellar and frontal eye field circuits responsible for smooth pursuit initiation and saccadic accuracy. This assessment was conducted at a single time point; repeat oculomotor testing was not performed during the treatment course.

#### Computerized dynamic posturography and balance assessment

Computerized dynamic posturography (CDP) sensory organization testing placed the patient at the 0th percentile for age-matched composite equilibrium norms. Condition-specific results were as follows: Condition 1 (eyes open, stable surface) 85th percentile; Condition 2 (eyes closed, stable surface) 60th percentile; Condition 3 (visual conflict, stable surface) 45th percentile; Condition 4 (eyes open, unstable surface) 10th percentile; Condition 5 (eyes closed, unstable surface) 5th percentile; Condition 6 (visual conflict, unstable surface) 0th percentile. The progressive deterioration with increasing proprioceptive and vestibular challenge indicates dysfunction of somatosensory, vestibular, and visual integration pathways. This assessment was performed at a single time point; it was not repeated during the follow-up period.

#### Heart rate variability analysis

The 24-h heart rate variability (HRV) monitoring revealed reduced time-domain metrics (standard deviation of NN interval (SDNN) 28 ms; normative range greater than 50 ms), reduced frequency-domain metrics (low frequency/high frequency (LF/HF) ratio: 0.8; normative range: 1.5–2.0), and reduced total power (380 ms^2^; normative range greater than 1,000 ms^2^). These findings indicate significant autonomic dysregulation with reduced parasympathetic tone. HRV parameters are influenced by age, physical fitness, hypertension, and pharmacological agents, and the absence of a pre-injury baseline limits the extent to which these values can be attributed solely to the MVC. These findings are consistent with patterns of autonomic dysfunction reported in PPCS [20].

#### Olfactory assessment

Standardized olfactory testing using the University of Pennsylvania Smell Identification Test (UPSIT) returned a score of 32/40 (normative range for age and sex-matched controls: 35–40), indicating mild hyposmia. Post-traumatic olfactory dysfunction has been reported in mTBI and attributed to shear injury to the olfactory fila at the cribriform plate or to orbitofrontal cortex involvement [13]. It should be noted that mild hyposmia is also a recognized age-related change and may be influenced by the patient’s medical history, such that attribution to the index injury alone is uncertain.

#### Standardized psychological and symptom assessments

Validated psychological and symptom instruments were administered at the evaluation on April 19, 2023. The Patient Health Questionnaire-9 (PHQ-9) returned a score of 14/27, indicating moderate depression. The Generalized Anxiety Disorder-7 (GAD-7) returned a score of 12/21, indicating moderate anxiety. The PTSD Checklist for DSM-5 (PCL-5) returned a score of 38/80, which exceeds the clinical threshold of 33. The Perceived Stress Scale (PSS) returned a score of 24/40, indicating high perceived stress. The Headache Impact Test-6 (HIT-6) returned a score of 58/78, indicating substantial headache impact. The Mini-Mental State Examination, Second Edition (MMSE-2) returned a score of 26/30, which falls at the lower end of the clinically normal range (standard threshold for further cognitive evaluation is typically set at 24/30 on this instrument). These psychological findings are noteworthy in their own right and are also relevant confounders: PHQ-9, GAD-7, and PCL-5 scores at these levels are independently associated with reduced HRV, olfactory sensitivity, and cognitive performance, and their contribution to the functional test results cannot be fully disentangled.

#### Longitudinal symptom tracking

CSC scores were recorded at eight time points across the treatment course (Table 1). The patient reached complete symptom resolution (CSC = 0) by July 2023, 17 weeks after injury. A stress-related exacerbation in August 2023 drove the score back to 72, followed by re-resolution to 0 by October 2023. By February 2024, the score had stabilized at 8, reflecting near-complete resolution at 52 weeks post-injury. A second MVC occurred in November 2024, but outcomes from that event are outside the scope of this report. The CSC provided the only longitudinal functional data in this case; none of the eight functional assessment modalities described above was repeated during the follow-up period.

**Table 1:** Longitudinal Concussion Symptom Checklist Scores Across the Treatment Course

Date	CSC total score	Clinical context
March 23, 2023	92	Initial presentation (2 days post-MVC)
April 18, 2023	69	4 weeks post-injury; active rehabilitation initiated
June 6, 2023	60	11 weeks post-injury; ongoing rehabilitation
July 18, 2023	0	17 weeks post-injury; complete symptom resolution
August 24, 2023	72	22 weeks post-injury; stress-related exacerbation (no new injury)
October 5, 2023	0	28 weeks post-injury; re-resolution following program intensification
January 16, 2024	12	43 weeks post-injury; maintenance phase
February 22, 2024	8	52 weeks post-injury; near-complete resolution at 12-month mark

CSC scores were recorded at eight time points from initial presentation on March 23, 2023 through the 12-month follow-up visit on February 22, 2024. The table presents the date of assessment, the total CSC score (maximum 132), and the clinical context at each time point. CSC: Concussion Symptom Checklist; MVC: motor vehicle collision.

#### Rehabilitation program

Following the comprehensive multimodal assessment, an individualized rehabilitation program was designed to address each identified deficit domain. The program comprised 10 modalities delivered in coordinated fashion over the treatment period. Vestibular rehabilitation therapy (VRT) employed adaptation, habituation, and substitution exercises targeting balance deficits and vestibulo-ocular reflex (VOR) dysfunction, progressing from static to dynamic environments. Oculomotor rehabilitation delivered targeted exercises addressing saccadic dysmetria, smooth pursuit deficits, and fixation instability using computerized visual feedback and progressive difficulty levels. Cognitive rehabilitation provided structured training targeting attention, processing speed, working memory, and executive function through computerized and therapist-directed tasks. Heart rate variability biofeedback (HRV-BF) used slow-paced diaphragmatic breathing at resonance frequency (approximately 0.1 Hz) to improve cardiac autonomic regulation and reduce physiological stress reactivity. Non-invasive transcutaneous auricular vagus nerve stimulation (taVNS) via earlobe electrodes was applied to modulate autonomic tone and support parasympathetic recovery; the evidence base for taVNS in PPCS remains limited to early-phase studies. Alpha-Stim cranial electrotherapy stimulation (CES) via earlobe clips was applied to address sleep disturbance, anxiety, and mood dysregulation; controlled evidence for CES in this context remains sparse. Exercise with oxygen therapy (EWOT), comprising structured aerobic exercise while breathing high-concentration oxygen (90–95%), was used to support cerebral oxygenation and neurometabolic recovery; this modality lacks rigorous controlled-trial evidence in concussion populations. Cervical spine rehabilitation employed manual therapy and therapeutic exercise targeting cervicogenic contributions to headache and vestibular symptoms, including proprioceptive retraining. Transcranial photobiomodulation (PBM, low-level laser therapy) was applied to support mitochondrial function and reduce neuroinflammation; the evidence base for this modality in PPCS is currently limited to small uncontrolled studies. Nutritional and supplementation support included omega-3 fatty acid supplementation, magnesium glycinate, and anti-inflammatory dietary modifications to support neurometabolic recovery and reduce oxidative stress.

## Discussion

### Diagnostic significance of multimodal assessment

Several clinically important observations emerge from this case. The central finding is that a patient with entirely normal standard neuroimaging harbored functional abnormalities across multiple domains on a comprehensive multimodal battery. This is consistent with the well-established limitation of conventional structural imaging in detecting the functional sequelae of mTBI, which prior literature has characterized in detail [3, 9, 10, 13]. This case should be read as an illustration of that principle rather than as novel evidence that conventional imaging misses PPCS physiology; that observation is already established in the literature.

The co-occurrence of abnormal findings across eight distinct assessment modalities is noteworthy. The VNG pattern of central vestibular dysfunction affecting pontine, midbrain, cerebellar, and parietal pathways is consistent with the known vulnerability of vestibulo-cerebellar circuits to diffuse axonal injury [7, 8]. The qEEG findings of reduced P300 amplitude and increased delta power corroborate evidence that cortical electrophysiology may be more sensitive than structural imaging in detecting functional consequences of mTBI [11]. Viewed together, these findings are consistent with the conceptual framework that PPCS reflects distributed neural network dysfunction rather than focal structural damage [3, 9, 10], and they are also interpretable within the NERD model of reflex circuit dysfunction in PPCS [12].

However, several important caveats bear emphasis. First, the sensitivity, specificity, and predictive values for PPCS have not been formally established for all of the modalities used here, including VNG, automated pupillometry, CDP, and the C3 Logix platform. The findings of each test are therefore best interpreted as clinically supportive rather than independently diagnostic. Second, each functional modality was assessed at a single time point only; none was repeated during follow-up. As a result, objective functional recovery across these circuits cannot be demonstrated from the available data, and neuroplasticity claims are appropriately limited to what the longitudinal symptom data support. Third, the convergence of abnormalities across tests reduces the probability that any single result is artifactual, but convergence does not rule out shared confounders. Age of 57, treated hypertension, ongoing medication use, and the documented psychiatric burden (PCL-5 above threshold, moderate PHQ-9, and GAD-7 scores) are each independently associated with HRV reduction, pupillary slowing, olfactory decline, and cognitive variability. The absence of any pre-injury functional baseline means that the extent to which each finding is attributable to the index injury, rather than to pre-existing factors, cannot be determined with certainty.

### Rehabilitation outcomes

The decline in CSC scores from 92 at initial presentation to 0 at 17 weeks, followed by near-complete re-resolution to 8 at 52 weeks, is consistent with outcomes documented in published multimodal concussion rehabilitation programs [14, 15]. This trajectory is encouraging and may reflect a benefit from the structured deficit-specific program; however, it must be interpreted with caution. Ten concurrent rehabilitation modalities were delivered over this period, which makes it impossible to attribute improvement to any single intervention or combination of interventions. The observed recovery trajectory is also consistent with natural recovery from mTBI, which is known to occur over this timeframe in a proportion of patients. For several of the specific modalities used, including Alpha-Stim CES, taVNS, EWOT, and transcranial PBM, the evidence base currently comprises early-phase or uncontrolled studies, and their specific contribution to outcome in this case cannot be established. The most defensible interpretation is that the structured, comprehensive program was associated with symptom improvement and that it may have facilitated the recovery process; causal attribution to specific elements is not warranted by the design.

The transient exacerbation in August 2023, with CSC rising from 0 to 72 without a new injury, is consistent with the phenomenon of functional vulnerability in recovering neural networks, whereby physiological or psychological stressors can temporarily disrupt circuits that have not fully consolidated recovery [20]. The subsequent re-resolution to 0 by October 2023 suggests that the rehabilitation gains were durable and that recovery capacity was retained.

### Clinical implications

This case carries several practical messages for clinicians managing patients with persistent post-concussion symptoms following normal neuroimaging. A comprehensive functional assessment battery incorporating VNG, qEEG, pupillometry, CDP, oculomotor tracking, HRV analysis, and computerized concussion assessment (such as C3 Logix) may document functional abnormalities that guide targeted rehabilitation planning, even in the absence of structural imaging findings. The NERD model framework provides a structured approach for interpreting multidomain functional findings and designing targeted rehabilitation interventions [12]. Longitudinal symptom tracking using a validated instrument such as the CSC is valuable for monitoring treatment progression and identifying exacerbations. The multimodal rehabilitation approach appears to offer a clinically meaningful benefit in this patient population, consistent with the emerging interdisciplinary concussion management literature [16–19], although controlled evidence for several individual modalities remains limited.

Regarding the time investment required for this comprehensive battery: the C3 Logix assessment was completed in approximately 28 min; the 24-h HRV monitoring was conducted at home as a continuous recording. The in-clinic components, including VNG, qEEG, pupillometry, CDP, oculomotor assessment, olfactory testing, and psychological instruments, collectively represent a multi-session assessment commitment. Clinicians considering adoption of this battery in high-volume settings should account for this time investment and may wish to prioritize or sequence assessments based on the individual patient’s symptom profile.

For patients with persistent post-concussion symptoms, the evidence from this case supports re-evaluation at clinically meaningful intervals rather than a single-point-in-time assessment. Given the exacerbation-recovery pattern observed here, at minimum, regular CSC monitoring at each clinical contact is warranted. Repeat functional testing at approximately 3-month intervals would allow for the tracking of objective recovery across modalities; further research is needed to determine optimal re-assessment intervals and to identify which modalities are most responsive to treatment-related change.

### Limitations and future directions

Several important limitations of this case report should be acknowledged. As a single case report, generalization to the broader PPCS population is inherently constrained, and the findings are not generalizable beyond the individual described. The absence of a pre-injury functional baseline is a fundamental limitation: without it, attribution of abnormal findings to the index MVC rests on temporal association and clinical plausibility rather than direct comparison. The concurrent psychiatric burden, hypertension, age-related changes, and medication use represent meaningful confounders for multiple test modalities, and their relative contributions cannot be quantified. The single-timepoint design for the functional assessment battery (with the exception of the CSC) means that objective recovery of the circuits assessed cannot be demonstrated from the available data; improvement in CSC scores alone does not establish neurophysiological recovery. The 10-modality rehabilitation program precludes attribution of benefit to any specific intervention. The reliance on proprietary normative databases for qEEG and BTA introduces a transparency limitation, as independent replication against open normative standards is not possible. No serum biomarker assessment was performed as part of this clinical workup; neurofilament light chain and tau/phosphorylated tau, which have demonstrated utility as chronic-phase biomarkers in the subacute to chronic post-injury period, were not obtained, and their absence represents an additional limitation of this evaluation. Finally, two of the references cited in this manuscript [9, 11] are currently available as preprints that have not undergone peer review, and the evidence they represent should be weighted accordingly.

Addressing these limitations in future work will require prospective, multicenter cohort designs that include pre-injury or matched-control baseline functional assessments, repeated-measures functional testing at standardized follow-up intervals, and statistical adjustment for relevant demographic and clinical confounders. Whether the full multimodal functional battery should be applied to pediatric concussion populations, in whom neurobiological vulnerabilities and recovery trajectories differ substantially from adults, is an open question that merits dedicated investigation. Future studies should also aim to identify which subset of the multimodal battery provides the highest diagnostic yield and the most responsive treatment monitoring signal, given the practical and resource constraints of clinical implementation.

### Conclusions

This case demonstrates that a comprehensive multimodal functional assessment can identify functional abnormalities consistent with mTBI pathophysiology even when conventional neuroimaging is entirely unremarkable, thereby supporting the clinical diagnosis of PPCS and enabling a targeted rehabilitation plan. The convergence of abnormalities across vestibular, electrophysiological, autonomic, oculomotor, postural, olfactory, and computerized concussion assessment domains provides a clinically meaningful picture that standard CT and MRI alone could not have established, while recognizing that none of these modalities has fully established diagnostic characteristics for PPCS and that important confounders were present. The subsequent multimodal rehabilitation program was associated with complete symptom resolution by 17 weeks, with durable improvement maintained at 52 weeks, although causal attribution to any specific intervention is not possible from a single uncontrolled case. These findings support further investigation into the routine integration of multimodal functional assessment into the clinical evaluation of patients presenting with persistent symptoms following mTBI.

## Data Availability

De-identified clinical records supporting the conclusions of this article are available from the corresponding author upon reasonable request.

## Abbreviations

BTA: brain timing assessment
CDP: computerized dynamic posturography
CES: cranial electrotherapy stimulation
CSC: Concussion Symptom Checklist
CT: computed tomography
cVEMP: cervical vestibular evoked myogenic potential
DMN: default mode network
DSM-5: Diagnostic and Statistical Manual of Mental Disorders, Fifth Edition
DTI: diffusion tensor imaging
EWOT: exercise with oxygen therapy
GAD-7: Generalized Anxiety Disorder-7
HIT-6: Headache Impact Test-6
HRV: heart rate variability
HRV-BF: heart rate variability biofeedback
LF/HF: low frequency/high frequency
mBESS: modified balance error scoring system
mTBI: mild traumatic brain injury
MMSE-2: Mini-Mental State Examination, Second Edition
MRI: magnetic resonance imaging
MVC: motor vehicle collision
NERD: neurological examination of reflex dysfunction
oVEMP: ocular vestibular evoked myogenic potential
PBM: photobiomodulation
PCL-5: PTSD Checklist for DSM-5
PHQ-9: Patient Health Questionnaire-9
PPCS: persistent post-concussion syndrome
PSS: Perceived Stress Scale
qEEG: quantitative electroencephalography
SAC: standardized assessment of concussion
SDNN: standard deviation of NN interval
SWI: susceptibility-weighted imaging
taVNS: transcutaneous auricular vagus nerve stimulation
UPSIT: University of Pennsylvania Smell Identification Test
VNG: videonystagmography
VOR: vestibulo-ocular reflex
VRT: vestibular rehabilitation therapy
